# An effective approach for identification of *in vivo *protein-DNA binding sites from paired-end ChIP-Seq data

**DOI:** 10.1186/1471-2105-11-81

**Published:** 2010-02-09

**Authors:** Congmao Wang, Jie Xu, Dasheng Zhang, Zoe A Wilson, Dabing Zhang

**Affiliations:** 1 School of Life Science and Biotechnology, Shanghai Jiao Tong University, Shanghai 200240, China; 2 School of Biosciences, University of Nottingham, Sutton Bonington Campus, Loughborough, Leicestershire, LE12 5RD, UK; 3 Bio-X Research Center, Key Laboratory of Genetics & Development and Neuropsychiatric Diseases, Ministry of Education, Shanghai Jiao Tong University, Shanghai 200240, China

## Abstract

**Background:**

ChIP-Seq, which combines chromatin immunoprecipitation (ChIP) with high-throughput massively parallel sequencing, is increasingly being used for identification of protein-DNA interactions *in vivo *in the genome. However, to maximize the effectiveness of data analysis of such sequences requires the development of new algorithms that are able to accurately predict DNA-protein binding sites.

**Results:**

Here, we present SIPeS (**S**ite **I**dentification from **P**aired-**e**nd **S**equencing), a novel algorithm for precise identification of binding sites from short reads generated by paired-end solexa ChIP-Seq technology. In this paper we used ChIP-Seq data from the *Arabidopsis *basic helix-loop-helix transcription factor ABORTED MICROSPORES (AMS), which is expressed within the anther during pollen development, the results show that SIPeS has better resolution for binding site identification compared to two existing ChIP-Seq peak detection algorithms, Cisgenome and MACS.

**Conclusions:**

When compared to Cisgenome and MACS, SIPeS shows better resolution for binding site discovery. Moreover, SIPeS is designed to calculate the mappable genome length accurately with the fragment length based on the paired-end reads. Dynamic baselines are also employed to effectively discriminate closely adjacent binding sites, for effective binding sites discovery, which is of particular value when working with high-density genomes.

## Background

DNA-binding proteins such as transcription factors (TFs), insulators or DNA modifying enzymes regulate various biological processes. Chromatin immunoprecipitation coupled with genome tiling microarrays (ChIP-chip) [1,2] and sequencing (ChIP-Seq) [3-6] have become important tools to systematically identify protein-DNA interactions. Particularly ChIP-Seq, which combines ChIP with massively parallel sequencing, offers a new genome-wide approach to extensively determine chromosome binding sites of DNA-associated proteins. However the massive amounts of data generated from the high-throughput sequencing pose great challenges for the identification of protein binding sites.

Several statistical approaches have been developed for analyzing ChIP-Seq data generated by single-end sequencing to find genomic regions that are enriched in a pool of specifically precipitated DNA fragments. These data can be used to determine the binding sites of TFs, using algorithms such as MACS, QuEST, SISSRs, ChIP-Seq processing pipeline, F-Seq, FindPeaks, ChIPDiff, CisGenome and PeakSeq [7-15]. These algorithms work in a similar way, in which the enriched regions are deduced through the calculation of the tag density in a window/bin of a certain size in the genome. An estimation of the fragment size is used, typically by extending the read lengths of their 3'ends to identify binding motifs in these algorithms [16]. However, uncertain prediction of the precise DNA-protein binding sites still occurs, thus ChIP-Seq analysis is recognized as a relatively immature technology which requires development [16].

The Paired-end Illumina sequencing platform is a recently emerging technology, which has been developed based on the single-end sequencing system. The paired-end sequencing system generates double-end sequencing reads using the Paired-End Module, which directs regeneration and amplification operations to prepare the templates for a second round of sequencing [17]. The double-end reads can be used for more precise identification of each corresponding DNA fragment; therefore the paired-end sequencing data has the potential to increase the accuracy of identification of chromosome binding sites of DNA-associated proteins because the fragment length as well as the effective genome length can be computed accurately.

Here we describe a novel algorithm, SIPeS (**S**ite **I**dentification from **P**aired-**e**nd **S**equencing), which can be used to effectively mine the paired-end sequencing reads for genome-wide identification of binding sites by calculating fragment pileup values (number of overlapping DNA fragments) at each nucleotide position. Then a dynamic baseline, a background model and other user-set thresholds are used to find the binding sites. We demonstrate the utility of this algorithm with a ChIP-Seq data set generated using the solexa platform for genome-wide binding analysis of a transcription factor ABORTED MICROSPORES (AMS). AMS belongs to a basic helix-loop-helix (bHLH) transcription factor, which is required for tapetal cell development and the post-meiotic microspore formation in *Arabidopsis thaliana *[18]. Using an *in vitro *selection and amplification binding assay, the recombinant AMS fusion protein was shown to bind to the 6-bp consensus bHLH binding DNA motif sequence CANNTG, typically referred to as the E-box [19]. The performance of SIPeS was compared to two algorithms, Cisgenome and MACS, used for reporting specific binding motifs and revealed that SIPeS has better resolution for binding sites discovery.

## Methods

### Chromatin Immunoprecipitation (ChIP)

The procedure for ChIP of AMS-DNA complexes in the wild-type *Arabidopsis *anther was modified from that of Saleh et al [20]. Chromatin was isolated from 1.5 g of formaldehyde cross-linked tissue from 0.6-1.1 mm buds of plants showing *AMS *expression [18]. For immunoprecipitation, we used a specific polyclonal AMS antibody, which in Western blot analysis, interacts exclusively with the AMS protein and shows no interaction with the *ams *mutant [19].

### Dataset

Since there is no public released paired-end ChIP-Seq data, we generated a ChIP-Seq library from an AMS IP sample using a specific AMS polyclonal antibody [19] on chromatin isolated from *Arabidopsis thaliana *buds. The aligned sequence reads for AMS are available in the Gene Expression Omnibus with accession number GSM424618. Library preparation, linker annealing, amplification, and gel purification for around 20 ng ChIP DNA were performed as instructed by the Illumina protocol with small modifications [17]. Gel purification and size selection for DNA fragments between 80 and 300 bp were done after the amplification step.

### Software availability

SIPeS is implemented in C and will be freely available for non-profit use of most genomes (i.e. human, mouse, rice). The source code and its executable file are available at http://gmdd.shgmo.org/Computational-Biology/ChIP-Seq/download/SIPeS. Users can compile SIPeS with the command line 'gcc -lm -O3 -s -g -o SIPeS SIPeS.c'. SIPeS runs from the command line and takes the following parameter: -bs for dynamic baseline start to construct the signal map, signal map means the picture of fragment pileup value at each nucleotide position; -be for dynamic baseline end to construct the signal map; -p for *p*-value cutoff to call peaks; -f for fold-enrichment to find signal maps based on the Poisson model.

Two types of files can be produced by SIPeS. One type generates signal coordinates and the pileup value of fragments from each chromosome. The other file includes signal start, signal end, signal width, reads in signal, max fragment pileup value, *summit *start, *summit *end, *summit *middle, *summit *width, *p*-value, and fold-enrichment of each signal map. Here, *summit *means the location with a single global maximum fragment pileup value in the signal map.

### Modeling the DNA fragments of paired-end reads

Paired-end sequencing technology generates large numbers of reads derived from both the 5' and 3' ends of fragmented DNA (here called end-1 and end-2) in a ChIP-Seq library. Using the Illumina Solexa platform 17.3 million 40-bp sequence reads were obtained from the AMS IP sample. From these data, 3,371,349 end-1 reads and 3,371,349 end-2 reads were uniquely mapped onto the *Arabidopsis *genome (Tair8) [21] by SSAHA2 (version 2.3) [22], allowing a maximum of one mismatch and no gaps in either end-1 or end-2 when the corresponding sequences mates aligned over a range of 80 to 500 bp. This process can also be used by other mapping software which supports the paired-end reads such as MAQ [23] and Bowtie [24].

**Figure 1:**
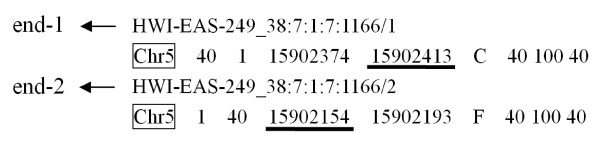
**Example of an extracted fragment's start and end position from SSAHA2 paired-end mapping data**. Extracting the minimum and maximum chromosome coordinate information from end-1 reads and end-2 reads. Here, 15902154 and 15902413 are the minimum and maximum coordinates, respectively, so the starting nucleotide for the reconstructed fragment is 15902154 and the end position is at 15902413 on Chr5.

Each pair of the sequenced reads is allocated a unique '*token*' (reading name), that allows the precise identification of each corresponding DNA fragment. Using the mapped paired-end reads coordinate chromosome information, the start and end position of each fragment can be easily extracted via the preprocessing program of SIPeS (Figure 1). The precise location of each fragment in the reference genome is determined and these fragments are used as the SIPeS input.

## Results

### Calculation of the effective genome size

The paired-end sequencing technology generates double-end reads with unique tokens that can be used to determine the DNA fragment's location and their corresponding length using SIPeS. Using the preprocessing program of SIPeS, the effective genome size, which is the genome coverage calculated based on uniquely mapped reads, within the *Arabidopsis *genome is 111,755,668 bp, which accounts for about 93% of the whole genome length in our AMS experiment (excluding chloroplast and mitochondrion genome sequence).

### SIPeS algorithm

**Figure 2:**
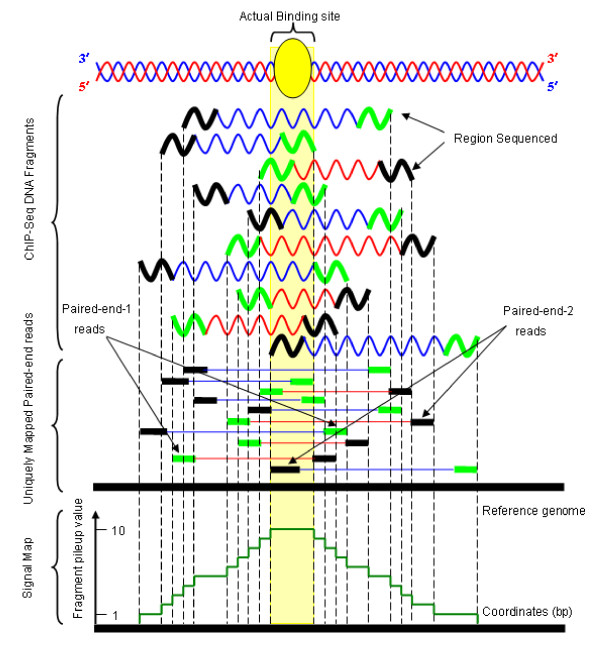
**Schematic overview of the SIPeS algorithm**. Sequenced short tags (here 40 bp) from paired-end sequencing are mapped onto the reference genome to find their corresponding aligned sequences or 'mates match' within a range of 80 to 500 bp. The fragment's start position and end position are then used to determine the putative binding sites.

**Figure 3:**
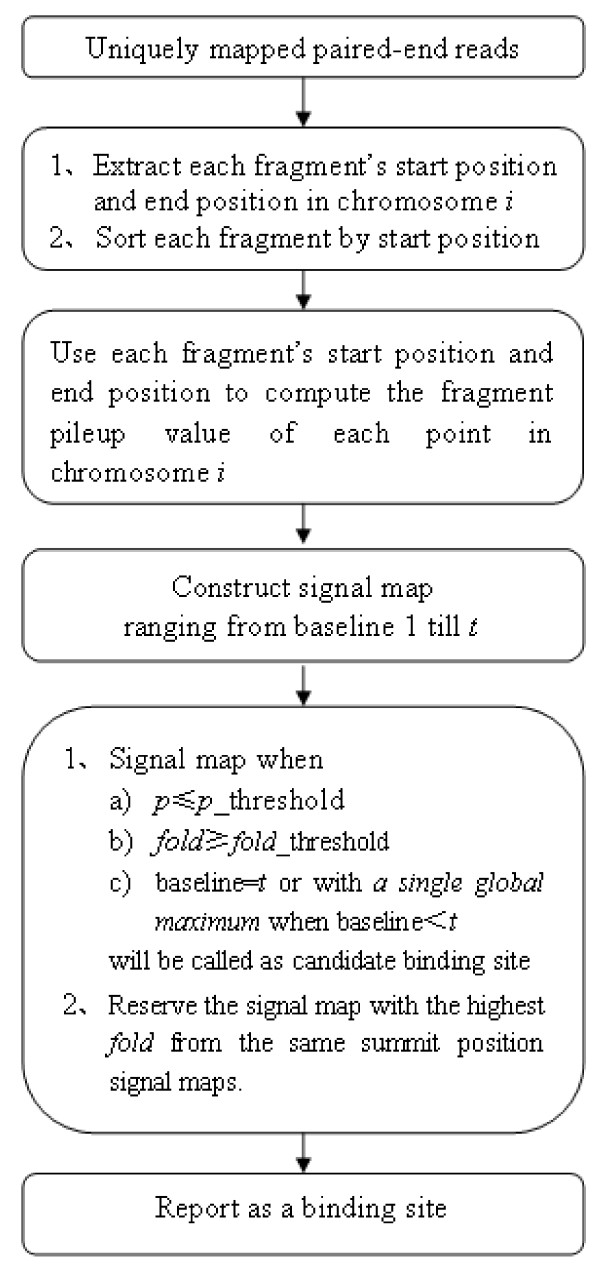
**Workflow of the SIPeS algorithm**. Using mapped paired-end reads information to compute the fragment pileup value for each point in chromosome *i*, then a signal map is constructed starting from baseline 1 until *t *(*t *= the maximum baseline to construct the signal map). Each signal map satisfies the user-set thresholds with a single global *summit *called as a candidate binding site when the baseline is smaller than *t*.

SIPeS uses the fragment's start position and end position to identify binding sites (Figure 2) and its algorithm is overviewed in Figure 3. Briefly, the fragments are extracted by using the start and end positions defined in 'chromosome *i*'. Then fragment pileup value is calculated based on the sorted fragments in 'chromosome *i*' (Additional file 1 for details). Subsequently, a dynamic baseline is used to cut off the bottom of peaks to identify potential binding sites. Here baseline means the fragment pileup value (Figure 2) when start to construct the signal map. SIPeS is able to construct signal maps ranging from baseline 1 to *t*; *t *refers to the maximum baseline which can be set by users. After all the signal maps are constructed, SIPeS evaluates whether each signal map satisfies a set of user-determined thresholds for finding the true binding events.

The probability of detecting a binding site within a signal width *w *supported by at least *c *reads by chance based on its baseline is given by a sum of Poisson distribution [5] as:

where *tpn *means the total number of paired-end reads (end-1, end-2), *gs *means the mappable genome size which is 111,755,668 bp in our AMS experiment, and the ratio between *c *and *w(tpn/gs) *reported as the fold-enrichment (*fold*).

SIPeS also allows users to use their input DNA as background. Then false discovery rate can be calculated using *n*1/*n*2, here *n*1 means the peak number that called by SIPeS when using ChIP over input DNA, and *n*2 means the peak number that called by SIPeS when using input DNA over ChIP with the same cutoff as *n*1. Then , where *i *means input DNA reads count in a signal width *w *and *c *means IP reads count in *w*, also *r *is the normalization ratio of total IP reads count and total input DNA reads count sequenced by the Illumina Solexa platform.

Candidate signal maps with *p *below a user-defined threshold *p*-value and *fold *above a threshold fold-enrichment are called if baseline = *t*, or the signal maps with a single global maximum when baseline <*t*. Since the signal maps are constructed ranging from baseline 1 to *t*, some candidate signal maps may have the same global maximum positions; SIPeS finally records a peak with the highest signal map value *fold *from the same 'global maximum position' as one binding site. And then all the called regions are ranked based on the *fold*.

### Method comparisons

SIPeS processes the data of paired-end sequencing by piling up the fragments in the genome. Although the updated MACS system supports the paired-end mode [7], current publically available ChIP-Seq algorithms are mainly targeting to single-end sequencing data. These existing ChIP-Seq peak finding methods generally predict peaks by estimating the fragment length to predict the peak shift and use tag density within a window when dealing with the sequencing data. Here we compared SIPeS analysis with two publicly available ChIP-Seq peak finding methods, Cisgenome [14] and MACS (version 1.3.6.1) [7]. The mapped 3,371,349 end-1 and 3,371,349 end-2 reads with the same effective genome size were used for analysis using Cisgenome and MACS software. The depth of AMS ChIP-Seq is comparable with those used by other algorithms such as 2.2 million ChIP tags for NRSF, 2.9 million for CTCF of human in MACS [7]. Moreover, human genome size is about 30 times larger than Arabidopsis.

**Figure 4:**
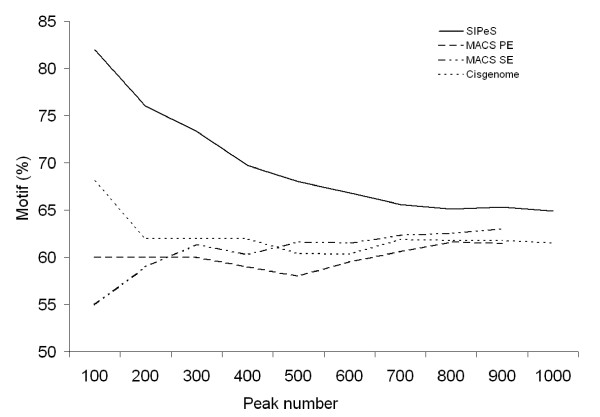
**Comparisons of the detection of the frequency of AMS binding sites**. The frequency of AMS binding motif occurrence within 200 bp from the AMS peak centers was compared using Cisgenome, MACS single-end mode (SE), MACS paired-end mode (PE) and SIPeS. From the smaller peak numbers to the higher, SIPeS generated a higher percentage of peaks containing the AMS binding motif than those of other three approaches.

**Figure 5:**
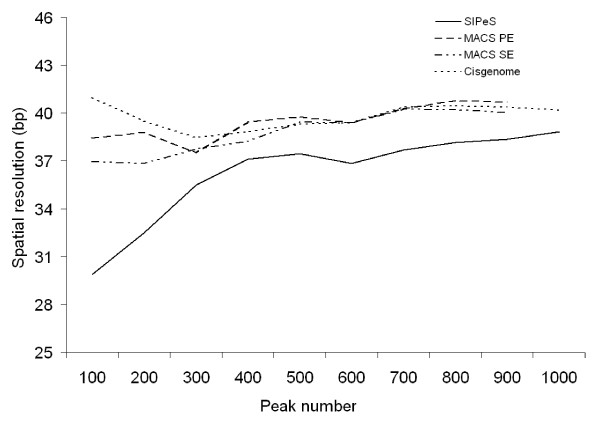
**Comparisons of the spatial resolution of detection of AMS binding sites**. Comparisons of the average distance (bp) from the peak center to the nearest motif (peaks with no motif within 200 bp from the peak center were removed) for AMS were made using Cisgenome, MACS single-end mode (SE), MACS paired-end mode (PE) and SIPeS. SIPeS displays a finer spatial resolution (bp) of peaks containing the AMS binding motif than those of Cisgenome and MACS single-end mode and MACS paired-end mode.

The data generated by MACS were compared with those of SIPeS using the single-end and paired-end modes of MACS. A total of 1,644 putative AMS binding sites were identified using SIPeS (*p *< 1 × 10^-5^, *fold *> 2), whilst only 954 binding sites were determined using the MACS paired-end mode (*p *< 1 × 10^-2^, *fold *> 1), and 981 binding sites using the MACS single-end mode (*p *< 1 × 10^-3^, *fold *> 1). We calculated the percentage of peaks harboring the AMS binding motif (CANNTG) within 200 bp (+/- 100 bp) of the peak center, and observed that SIPeS generated a higher percentage of peaks containing the AMS binding motif than those of Cisgenome, MACS single-end mode and MACS paired-end mode (Figure 4). The spatial resolution is expressed by the average distance from the peak center to the nearest AMS motif. We observed that the average distance from the peak center to the nearest AMS motif by SIPeS (excluding the peaks with no motif within 200 bp of the peak center) was shorter than those of Cisgenome, MACS single-end mode and MACS paired-end mode, suggesting that SIPeS system has a better spatial resolution (Figure 5). Collectively, these results suggested that SIPeS is able to find peaks with better resolution for binding motif discovery.

## Discussion

Effective genomic size is an important parameter for the calculation of *p*-value, and the fact that the SIPeS analysis can calculate fragment length and effective genomic size accurately using the reads from paired-end sequencing means that it can provide enhanced identification of DNA-protein binding sites from ChIP-seq data. Currently, most of the available algorithms can not accurately calculate the fragment size because they are mainly designed for single-end ChIP-Seq data analysis, which usually uses the direction of reads to estimate the fragment length to identify binding sites [16]. The paired-end sequencing technology generates double-end reads with unique tokens that can be used to calculate fragment length using SIPeS. Moreover, SIPeS can calculate the accurate effective genomic size using the advantage of the accurate fragment length. Other algorithms, such as MACS recommend that the effective genome size of hg18 is about 90% of the whole genome length [7] while SISSRs recommends about 80% [9] and FindPeaks suggests about 70% [12]. However this estimation is likely to affect the accuracy of the analysis for researchers who use the ChIP-Seq technology. In this study, an effective genomic size of 111,755,668 bp of AMS enriched DNA was observed using the SIPeS preprocessing program, which accounts for approximately 93% of the *Arabidopsis *whole genome length. In addition, SIPeS calculates the DNA-protein binding sites on basis of the analysis of fragment pileup which is more intuitive and creditable, while most of the existing algorithms are based on the tag counts to test the enrichment.

Currently, most peak finding methods often employ a window scan for the whole genome with a step to calculate the read count and see if that can satisfy the statistical tests. Varying window size and step length may therefore cause differences in the results. SIPeS can determine peak end and start positions based on a dynamic baseline while other algorithms sometimes incorrectly split a true peak into two or more peaks. In addition, SIPeS uses a dynamic baseline to discriminate closely adjacent binding sites to easily separate adjacent overlapping peaks. For example, if a baseline of 1 is used, two closely adjacent signal map A and map B are misrepresented as a single peak (Additional file 2a - baseline 1, signal map C identified). But if a higher baseline is adopted, map A and map B are identified (Additional file 2a - baseline 2, signal maps A and B identified). SIPeS can also analyze broad peaks with high signal levels (i.e. 1 peak) while a peak of the same shape but of lower signal value with low signal values would have every local maxima (i.e. multiple peaks). For example, one peak with the summit 1 will be called when the baseline is below 10 and satisfies the *p*-value cutoff set by the user. When the baseline is increased to 10, then two peaks, one merging peak (1 and 2) and peak 3 will be called. When the baseline is increased to 12, three peaks (1, 2, 3) will be called (Additional file 2b). If the low signal value is not high enough to satisfy the *p*-value cutoff, then only broad peaks with higher signal will be called.

**Figure 6:**
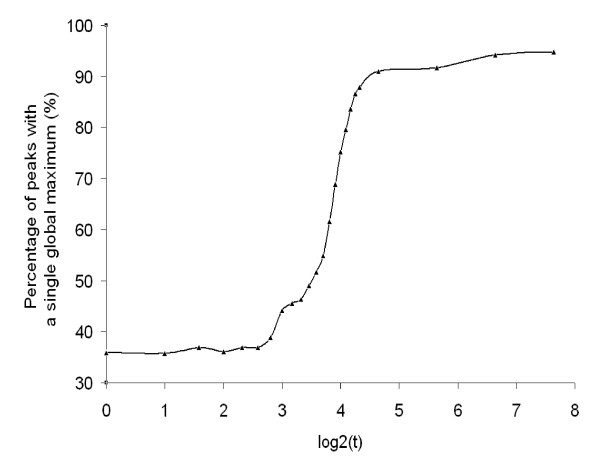
**Relationship between the maximum dynamic baseline *t *and percentage of signal maps with a single global maximum (*p *< 1 × 10^-5^, *fold *> 2) for AMS using SIPeS**. The percentage of peaks with a single global maximum appears increased when the *t *value is increased from the lower to the higher values. This suggests that SIPeS is able to effectively discriminate adjacent binding sites by increasing the *t *value.

**Figure 7:**
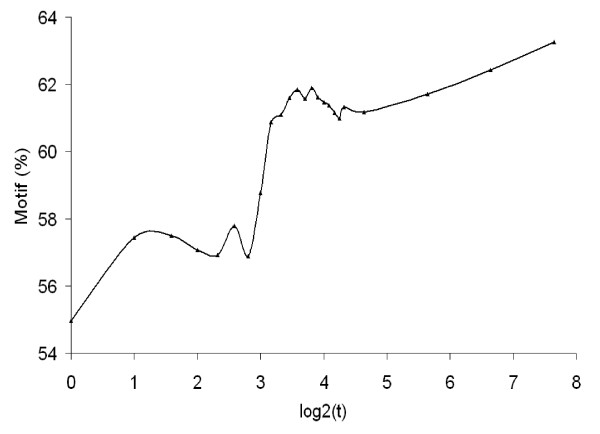
**Relationship between the maximum dynamic baseline *t *and percentage of AMS motif occurrence (*p *< 1 × 10^-5^, *fold *> 2) using SIPeS**. When *t *is increased from the lower to the higher, more AMS motif occurrence percentage is revealed, suggesting that *t *is a good indicator of finding binding sites.

**Figure 8:**
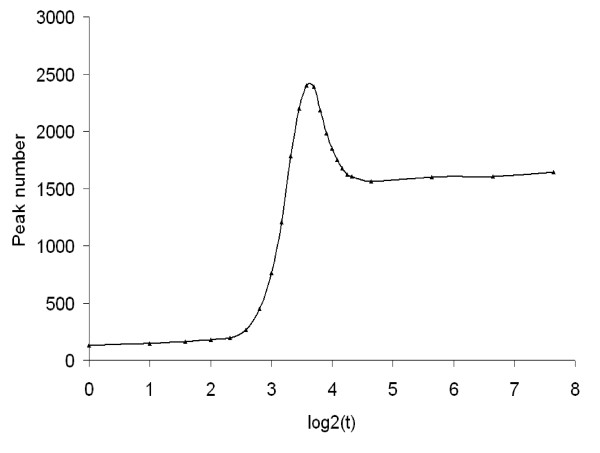
**Relationship between the maximum dynamic baseline *t *and the peak number for AMS (*p *< 1 × 10^-5^, *fold *> 2) called by SIPeS**. As *t *is increased from the lower to the higher, more peaks are called by SIPeS using the AMS paired-end ChIP-Seq reads, and peak number tends to be stable when *t *was increased from 25 to 200. This indicates that more genuine DNA-protein binding sites can be revealed by SIPeS.

Therefore by utilizing a dynamic baseline, SIPeS can theoretically find all the signal maps with a single global maximum (Figure 6), this is of particular importance for high-density genomes which may have a number of binding sites in close proximity. We found that motif occurrence percentage is higher when *t *is increased from 1 to 200 which mean peak results will be better with a high *t *value; suggesting *t *is a good indicator of finding binding sites (Figure 7). Also, peak number tends to be stable when *t *is increased using SIPeS, therefore users can find more genuine DNA-protein binding sites by increasing the *t *value (Figure 8). From analysis of our AMS ChIP-Seq data, we recommend the users to choose as high a value for *t *as possible since this will allow the peaks to be identified more accurately, even though it may take more time to achieve this goal. At the same time, SIPeS is able to report the percentage of peaks with a single global maximum based on *t *set by users which can judge whether *t *is set reasonable.

Similar to the limitation of existing algorithms, SIPeS is not suitable for peak finding in a wide peak region such as those histone marks, since the statistical tests are not capable of satisfying the user's threshold (for example, *p *< 0.01). Additionally, SIPeS algorithm is targeting to paired-end sequencing reads, and not applicable for single-end sequencing data.

## Conclusions

In this paper we present an algorithm SIPeS that can be used for calculation of the effective genome size and precise identification of binding sites from short reads generated from paired-end solexa ChIP-Seq technology. In comparison with two existing algorithms Cisgenome and MACS, we conclude that SIPeS has better resolution for binding sites identification. Moreover, the dynamic baseline used in SIPeS can effectively discriminate between closely adjacent DNA-protein binding sites, which is of particular value when working with high-density genomes.

## Authors' contributions

CMW and JX contributed equally to this work. CMW and DBZ conceived the strategies. DBZ supervised the project. CMW built the software. JX, DSZ and ZAW performed AMS ChIP and data analysis. DBZ, CMW, JX and ZAW wrote the paper.

## Supplementary Material

Additional file 1 SIPeS algorithm for calculating fragment pileup value after sort fragments by start position on chromosome *i*. Click here for file

Additional file 2 **Signal map with fragment pileup value determination using a dynamic baseline in SIPeS**. (a) When the baseline is below 2, one peak C would be observed by SIPeS, when the baseline is 2, peak A and peak B are observed. This scheme shows that SIPeS has the ability to accurately locate the DNA-protein binding sites using the dynamic baseline.(b) One peak with the summit 1 will be called when the baseline is below 10 and satisfies the *p*-value cutoff set by the user. When the baseline is increased to 10, then two peaks, one merging peak (1 and 2) and peak 3 will be called. When the baseline is increased to 12, three peaks, (1, 2, 3) will be called. Click here for file
