# Supplementary figures and images for: An effective approach for identification of in vivo protein-DNA binding sites from paired-end ChIP-Seq data
Source: BMC Bioinformatics. 2010 Feb 9;11:81. doi: 10.1186/1471-2105-11-81 (PMC2831849; doi:10.1186/1471-2105-11-81)

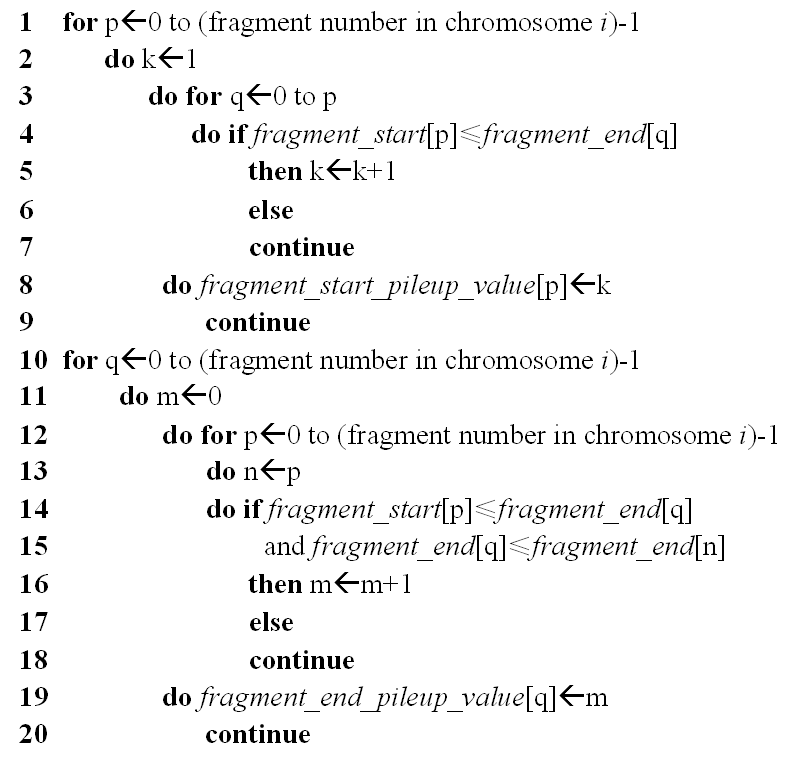

Supplement: Additional file 1 — SIPeS algorithm for calculating fragment pileup value after sort fragments by start position on chromosome i. [file 1471-2105-11-81-S1.DOC]

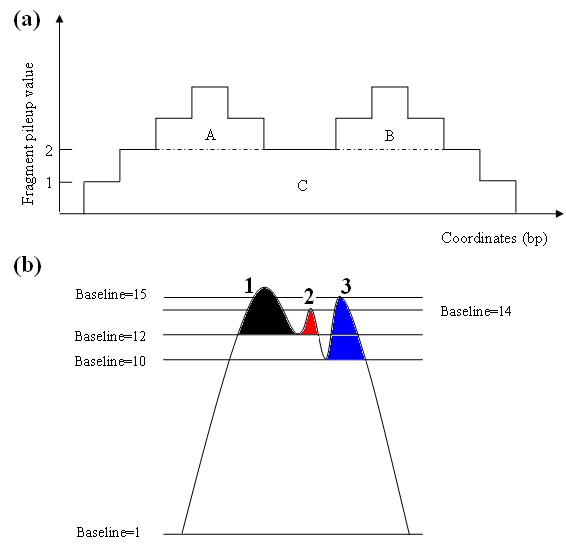

Supplement: Additional file 2 — Signal map with fragment pileup value determination using a dynamic baseline in SIPeS. (a) When the baseline is below 2, one peak C would be observed by SIPeS, when the baseline is 2, peak A and peak B are observed. This scheme shows that SIPeS has the ability to accurately locate the DNA-protein binding sites using the dynamic baseline.(b) One peak with the summit 1 will be called when the baseline is below 10 and satisfies the p-value cutoff set by the user. When the baseline is increased to 10, then two peaks, one merging peak (1 and 2) and peak 3 will be called. When the baseline is increased to 12, three peaks, (1, 2, 3) will be called. [file 1471-2105-11-81-S2.DOC]
